# The Medium-Term Outcomes of Patients With Suspected Scaphoid Fractures: A Single-Centre Retrospective Cohort Study

**DOI:** 10.7759/cureus.53361

**Published:** 2024-02-01

**Authors:** Chinmay Tijare

**Affiliations:** 1 Orthopaedics, University of Leicester, Leicester, GBR

**Keywords:** pain, patient reported outcome measures, outcomes, soft tissue injury, suspected scaphoid fracture

## Abstract

Background

The medium-term outcomes of patients (six to 14 months post-injury) with non-specific wrist injuries managed as suspected scaphoid fractures are not clear from the current literature. These patients’ wrists are immobilized in casts or splints, and some receive physiotherapy. They receive serial imaging and follow-up appointments as needed.

Aims

This study aims to describe the medium-term outcomes of patients with non-specific wrist injuries managed as suspected scaphoid fractures.

Methods

This is a single-centre retrospective cohort study. Patients with suspected scaphoid fractures were identified from a consecutive database and were included. Patients diagnosed with a definitive scaphoid fracture at any point in time were excluded. Patients with any pre-existing wrist pathology were also excluded.

In total 113 patients were posted the Patient-Rated-Wrist-Evaluation (PRWE) questionnaire at six to 14 months post-injury with a self-addressed return envelope. Demographic and PRWE data were collated and described.

Results

Twenty-two patients (19% of total patients) returned a completed questionnaire. The median PRWE score was 32 out of 100 indicating mild pain and disability. 45.5% of patients were in this category. A minority of patients (9%) continued to suffer severe or very severe pain and disability. Patients with PRWE scores <40, representing pain and disability that is mild or less, reported very low difficulty completing work and recreational activities. Patients tended not to have pain at rest and experienced the most difficulty lifting heavy objects.

Conclusion

Most patients with non-specific wrist injuries managed as suspected scaphoid fractures experience some pain and disability in the medium term. For most this is minimal or mild, however some patients experience significant pain and disability. This study adds to existing evidence that this is the case. The reasons why these patients suffer are unclear. This study highlights the need to refine clinical practice to improve the outcomes of these patients.

## Introduction

Injuries of the wrist are a common complaint in patients presenting to the emergency department [1]. Scaphoid fractures are the most frequently fractured carpal bone representing approximately 70% of all carpal fractures [2].

Clinical examination does not reliably diagnose scaphoid fractures [3]. Meta-analysis of 11 studies consisting of 1939 patients who had a positive clinical examination for a scaphoid fracture found that only 405 of these truly had a fracture giving a mean positive predictive value of under 21% [4]. 

Following clinical suspicion, radiographs are used to aid in the diagnosis of a scaphoid fracture. However, 30% of these fractures are not detectable on initial radiographs [5]. To avoid the consequences of an untreated occult scaphoid fracture, all patients with clinical signs of a scaphoid fracture but normal radiographs, in the absence of any other obvious pathology are treated as having a suspected scaphoid fracture.

Usual management of patients with suspected scaphoid fractures involves immobilisation in a cast or splint and further imaging to either exclude or confirm a definite fracture [6].

Literature search revealed only one study investigating the medium-term outcomes of these patients; this was a secondary outcome measure. Tiel-van Buul et al. [7] studied 28 patients being managed as having suspected scaphoid fractures. They were followed up one year after their injury and their symptoms were categorised into mild, moderate, or severe. Eighteen percent were found to have significant symptoms and grip strength was reduced in 36% of patients. In addition to the small sample size, this study is limited as grouping of symptoms is arbitrary and grip strength was the only measure of functional outcome.

A 2009 survey [8] representing almost 50% of the UK’s acute trusts demonstrated that there is substantial variation in the management of suspected scaphoid fractures. Similarly, a 2016 retrospective review [9] representing 141 acute trusts in England support these findings as it too found great variation in the management of patients with suspected scaphoid fractures. This includes variation in the use of imaging, physiotherapy, and types of immobilization methods.

A 2016 pilot study [10] compared patients receiving an early MRI scan to those receiving only plain radiographs initially. Pain and function scores were measured with the patient-reported wrist evaluation score (PRWE) as a secondary outcome at six-week follow-up and were found to be an average of 26 out of 100 in the group of patients with unconfirmed fractures (a score of 0 indicates no pain or disability and a score of 100 indicates the highest level of pain and disability). This provides some idea of the short-term outcomes, but this is too early to determine a meaningful effect of management for many patients.

Overall, there are no studies comprehensively describing the outcomes of patients with these wrist injuries.

Aim

This study aims to determine the medium-term outcomes of pain and function of patients with suspected scaphoid fractures. This will be measured using the PRWE.

## Materials and methods

This is a single-centre retrospective cohort study. A consecutive database of patients who underwent a scaphoid series of radiographs from the emergency department or urgent care centre associated with Nottingham University Hospitals (NUH) NHS Trust from 6th May 2020 to 5th November 2020 was reviewed.

The clinical notes of each patient were obtained and then reviewed to find those who were at first impression thought to have been treated as having a suspected scaphoid fracture by the specialist hand clinic at NUH. Clinical signs of a scaphoid fracture include anatomical snuffbox tenderness, scaphoid tubercle tenderness, pain with scaphoid compression test, and pain in snuffbox on wrist pronation followed by ulnar deviation [1]. The following inclusion and exclusion criteria in Table 1 were applied.

**Table 1:** Eligibility criteria

Inclusion	Exclusion
Patients aged over 16 at the time of injury.	Patients diagnosed with a definite scaphoid fracture at any point in time
Presenting with one or more of the clinical signs of a scaphoid fracture.	Patients with any additional wrist injury in the last six months
Seen in hand fracture clinic and managed as a suspected scaphoid fracture (clinical suspicion dictated by clinician, patient immobilized in a cast or splint and received further imaging at two weeks).	Patients with any co-existing wrist pathology

Demographic data of patients was also collected. This included patient names, addresses, sex, age, and length of follow-up measured from the initial presentation.

Patients were sent the PRWE questionnaire with a self-addressed envelope for them to return the completed questionnaire and a cover letter detailing the research project obtaining consent for the participants to be involved.

Approval

This study was registered as a service evaluation and approved in accordance with our local governance framework with the audit number 21-305C. All data was pseudonymised by creating a unique ID for each patient, encrypted, and held on the institutional OneDrive.

Statistical analysis

Pain and function scores were noted for each patient, these are equally weighted, and used to calculate the total PRWE score as described in the PRWE handbook [11]. Missing data was replaced with the mean of the subscale.

The overall PRWE scores were provided qualitative descriptors as described by MacDermid et al. [12] and histograms representing these scores were used to describe the distribution and skew of the data. Data analysis and graph generation were performed using Statistical Package for Social Sciences (SPSS) version 27 (IBM Corp., Armonk, NY, USA).

## Results

A total of 811 patients were to have had a scaphoid series of radiographs from 6th May to 5th November 2020. This meant that patients were being followed up six to 14 months after their injury. Three hundred seventy-six of these were noted to be treated as suspected scaphoid fractures. The eligibility criteria were applied, and this left 113 patients to be included in the study. These patients were sent the PRWE and their medical and demographic were noted for analysis. A total of 22 (19.5%) of patients returned completed questionnaires; there was no missing data in the completed questionnaires.

Demographics

The demographic data for these patients is shown in Table 2.

**Table 2:** Demographics

Variable	Value
Median Age (range)	40 (18-89)
Median Length of follow-up in days (range)	22 (14-232)
Number of Male respondents	4
Number of Female respondents	18

Pain and disability of patients was grouped according to severity as per the MacDermid et al. classification (Table 3) [12]. The median total PRWE score of 32 indicates mild pain and disability overall.

**Table 3:** Severity of pain and disability

Severity of Pain and Disability (using the MacDermid et al. classification [12])	Number of Patients	% of total patients
None	4	18.2
Minimal	3	13.6
Mild	10	45.5
Moderate	3	13.6
Severe	1	4.5
Very Severe	1	4.5

**Figure 1:**
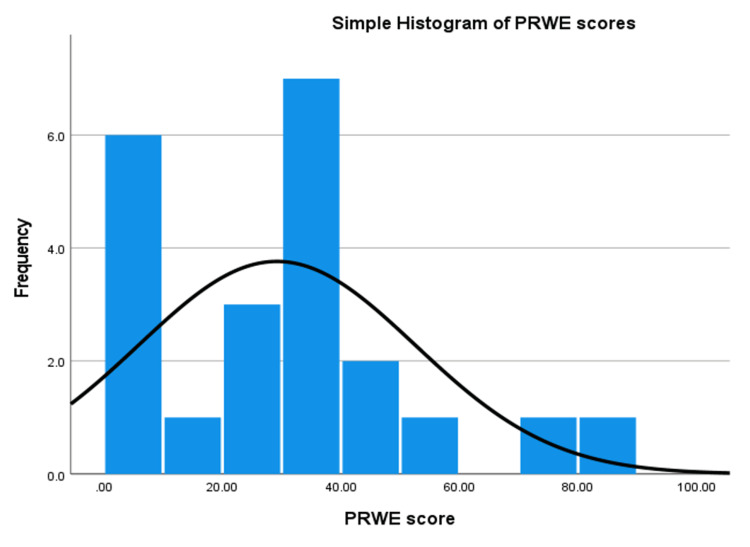
Histogram demonstrating the spread of total PRWE scores PRWE - patient reported wrist evaluation

The histogram (Figure 1) summarising the spread of the total PRWE demonstrates a non-normal distribution with a right skew of the data. Four patients had a PRWE score of 0 on follow-up, the maximum PRWE score was 81.50 and this patient had the highest pain and function scores of 43.00 and 38.50, respectively.

Table 4 describes the responses for individual items of the PRWE pain subscale at medium-term follow-up. The overall median pain score of 17.00 indicates mild pain. On average, patients had no pain at rest but had moderate pain when lifting heavy objects.

**Table 4:** Items within the pain subscale. IQR - interquartile range Scores are based on a linear scale from zero to 10. A score of zero indicates no pain when doing the task, whilst a score of 10 indicates the worst ever pain when doing the task

Pain (No pain – worst ever) (0-10)	At Rest	Repeated wrist movement	Lifting Heavy object	At its Worst	Frequency (Never-Always)	Pain Score (/50)
Median	0.00	2.00	5.00	5.00	3.00	17.00
IQR	1.00	6.00	8.00	8.00	2.00	23

Table 5 describes these items for the function subscale as well as the total PRWE score. The results indicate that on average, patients experienced mild functional disability when performing specific activities and minimal functional disability when performing usual activities. 

**Table 5:** Items within the function subscale IQR - interquartile range, PRWE - patient reported wrist evaluation Scores are based on a linear scale from zero to 10. A score of zero indicates no difficulty doing the task, while a score of 10 indicates being unable to do the task.

Function (No difficulty – unable to do) (0-10)	Turning doorknob	Cutting meat	Fastening Buttons	Pushing up from chair	Carrying 10lb object	Using bathroom Tissue	Personal Care	Household Work	Work	Recreational Activities	Specific Activities (/60)	Usual Activities (/40)	Total Function	Total PRWE score (/100)
Median	1.00	1.00	1.00	2.00	2.00	1.00	1.00	2.00	2.00	2.00	14.00	7.00	10.50	32.00
IQR	6.00	3.00	2.00	5.00	8.00	1.88	3.00	4.00	6.00	5.00	19.00	20.00	20.00	35.00

## Discussion

Our findings demonstrate that most patients (81.8%) do report some pain and disability in the medium term. For the majority, this is minimal (13.6%) or mild (45.5%). This information is summarised in Table 3 and demonstrated by the right skewed histogram (Figure 1). Patients generally do not experience pain at rest and suffer the most pain when lifting a heavy object. Usual function was most severely disrupted in the domains of recreational activities, work, and household work.

Some patients continue to experience significant pain and disability; 13.5% of patients reported moderate, and one patient each reported severe and very severe pain and disability respectively. These patients had not returned to clinic for further evaluation nor were there any failed discharges. Therefore, there is no clear indication as to why these patients suffer poorer outcomes.

Some of the findings of our study agree with those of Tiel-van Buul et al. [7]. This is the only other published literature reviewing the medium-term outcomes of this group of patients. Both studies demonstrate that most patients do not suffer significant levels of pain and disability in the medium term, they tend to experience mild symptoms. However, the median PRWE score of our patients is higher than the normative median and mean PRWE scores (0 and 7.7 respectively) [13].

Thirty-six percent of the patients in Tiel-van Buul et al. reported a significant loss of grip-strength on follow-up [7]. In our study patients reported lifting and carrying objects to be the most difficult tasks. This indicates that loss of strength is a common problem in patients. This could be responsible for much of the reported disability that patients experience, evidence has shown grip strength to be an important predictor of disability [14].

Tiel-van Buul et al. [7] showed a greater proportion of their patients (39%) to have no symptoms than in our study (18.2%). This disparity could be explained by the arbitrary grouping of patient symptoms by Tiel-van Buul et al. Our findings show that the average patient does not experience no pain at rest and so if the researchers in this study had not asked specific questions as the PRWE does, symptoms may have been missed. It is important to note that both studies are limited by a small sample size.

Both studies showed that some patients continue to have significant pain and disability in the medium term. It is not completely clear why some patients experience poorer outcomes.

Table 4 and Table 5 demonstrate that patients without significant levels of pain and disability report lower PRWE scores across all the domains shown. Importantly it shows that, on average, these patients reported no difficulty in completing their work (median score of 0) and minimal difficulty (median score of 1) in completing recreational activities. From this, it could be speculated that some patients participate in work and recreational activities that are more demanding on the wrist and this causes them to experience higher levels of pain and disability which carries into the other domains.

It could also be speculated that these patients have suffered more serious soft-tissue injuries such as extrinsic or intrinsic carpal ligament ruptures which take a longer time to heal than other soft tissue injuries [15]. These pathologies may have shown up on additional imaging, however, there was no indication for this as patients were well at discharge and did not request further follow-up.

Kelson et al. [10] studied a group of patients who were managed as having a suspected scaphoid fracture and found their median PRWE score to be 26 (representing mild pain and disability) at six weeks with the highest PRWE score being 37.25. This study is also limited by a very small sample size. Patel et al. [16] reviewed pain outcomes of patients with suspected scaphoid fractures at six weeks post-injury using a Visual Analogue Scale (VAS) from 0 to 10. The researchers found the mean pain score to be 2.7 at a six-week follow-up. Although not directly comparable to PRWE outcomes, this does indicate a low level of pain at six weeks. The average length of follow up for our patients was three weeks and at the point of discharge, all patients were clinically well.

This evidence suggests that patients have better outcomes in the short term than in the medium term. It is likely that patients appear clinically well after a short period of rest and immobilization but on return to normal activity after this period, their symptoms worsen again.

Bialocerkowski [17] conducted a qualitative study interviewing patients suffering from various wrist injuries. Notably, patients described a varying degree of compensatory mechanisms. Some patients would use their non-affected hand to do most of their activities whilst others would still use their affected hand which affected their perception of their functional abilities. The work and recreational activities of patients may also affect their ability to compensate. These factors could contribute to the large variance (interquartile range (IQR)) in PRWE scores seen in our study.

Limitations

The results of the study are limited by retrospective data and a low response rate. The PRWE score of patients was only recorded at one point in time, which makes it impossible to account for the differences in perception between patients. There was a female-heavy bias in the respondents which makes the results less generalisable. There was no data available on which of the patients received physiotherapy and which patients had good compliance with it, which is a possible confounding factor.

Implications

This is the first study to use patient reported outcome measures to investigate the pain and functional outcomes of patients with suspected scaphoid fractures in the medium term. It brings to light a group of patients who still suffer pain and disability, though our patient numbers have not allowed us to draw significant conclusions. Importantly, this study highlights that it is a potential problem.

## Conclusions

Most patients with non-specific wrist injuries managed as suspected scaphoid fractures experience pain and disability in the medium term. For most this is minimal or mild, however some patients experience significant pain and disability. This study adds to existing evidence that this is the case. The reasons why these patients suffer are unclear.

Future work should aim to verify our findings in a larger population as well as investigate the reasons for some patients suffering pain and disability in the medium term. Understanding the cause of this would help lead to effective management of these patients.
